# Economic evidence on integrated care for stroke patients; a systematic review

**DOI:** 10.5334/ijic.847

**Published:** 2012-10-01

**Authors:** Johanneke F.M.M Tummers, Augustinus J.P Schrijvers, Johanna M.A Visser-Meily

**Affiliations:** Master of Public Health, Julius Centre for Health Sciences and Primary Care, University Medical Centre Utrecht, Kanaalstraat 221BIS, 3531 CH Utrecht, The Netherlands; Professor of Public Health, Julius Centre for Health Sciences and Primary Care, University Medical Centre Utrecht, UMC Utrecht, div. Julius Centrum, Huispost Str. 6.131, Postbus 85500, 3508 GA Utrecht, The Netherlands; Rehabilitation specialist, University Medical Centre Utrecht, Rudolf Magnus Institute of Neuroscience and Centre of Excellence for Rehabilitation Medicine, University Medical Centre Utrecht and De Hoogstraat, Utrecht, The Netherlands. Address: UMC Utrecht, Huispost F00 810, Postbus 85500, 3508 GA Utrecht, The Netherlands

**Keywords:** stroke, integrated care, rehabilitation, costs, economics, review

## Abstract

**Introduction:**

Given the high incidence of stroke worldwide and the large costs associated with the use of health care resources, it is important to define cost-effective and evidence-based services for stroke rehabilitation. The objective of this review was to assess the evidence on the relative cost or cost-effectiveness of all integrated care arrangements for stroke patients compared to usual care. Integrated care was defined as a multidisciplinary tool to improve the quality and efficiency of evidence-based care and is used as a communication tool between professionals to manage and standardize the outcome-orientated care.

**Methods:**

A systematic literature review of cost analyses and economic evaluations was performed. Study characteristics, study quality and results were summarized.

**Results:**

Fifteen studies met the inclusion criteria; six on early-supported discharge services, four on home-based rehabilitation, two on stroke units and three on stroke services. The follow-up per patient was generally short; one year or less. The comparators and the scope of included costs varied between studies.

**Conclusions:**

Six out of six studies provided evidence that the costs of early-supported discharge are less than for conventional care, at similar health outcomes. Home-based rehabilitation is unlikely to lead to cost-savings, but achieves better health outcomes. Care in stroke units is more expensive than conventional care, but leads to improved health outcomes. The cost-effectiveness studies on integrated stroke services suggest that they can reduce costs. For future research we recommend to focus on the moderate and severely affected patients, include stroke severity as variable, adopt a societal costing perspective and include long-term costs and effects.

## Introduction

Annually, 15 million people worldwide suffer from a stroke. Of these, five million die and another five million become permanently disabled [1]. Many persons who have survived a stroke experience problems in one or more health-related domains, such as physical, cognitive, behavioural and emotional well-being. With an aging population and improved treatment options, this number is only expected to increase. In many Western countries, stroke is the biggest single cause of disability [1]. It should not come as a surprise that the economic burden of stroke is very large. Apart from the costs to the health system and rehabilitation services, there are also the production losses to society rising from premature deaths and disability, and costs to the individual and families who have to take time off from work to provide informal care. The extent of the problem, combined with limited health care budgets emphasizes the need for an evidence-based and cost-effective health care delivery in stroke care.

## Theory

Stroke care is complex and covers a whole spectrum of care including acute care, rehabilitation and long-term care with both in-hospital, outpatient and community-based care. Because of all these different types of care; stroke care is per definition multidisciplinary and the integration of care is of particular concern [2]. Ideally, optimal stroke care integrates all relevant care providers; hospitals, nursing homes, rehabilitation centres, general practitioners and home care providers who work together to provide multidisciplinary, coordinated care through organized patient transfers and protocols. In the last years, there has been a call to improve health care delivery to patients in terms of greater consistency, effectiveness, care continuity and improved collaboration [3].

In light of these developments, various ways of organizing and delivering care for stroke patients have been implemented. In this review, we focus on all stroke care *beyond* emergency (pre-)hospital care. Two Cochrane reviews concluded that more organized inpatient care for stroke patients was consistently found to yield better health effects [4, 5]. However, it remains unclear which way of delivering and coordination of comprehensive and multidisciplinary post-emergency care along the disease continuum and across the different health care systems is the most cost-effective.

Previous reviews into the cost-effectiveness of integrated stroke care have looked at only one type of integrated care arrangement, or looked only at in-hospital costs [4, 6, 7]. When studying integrated care interventions for stroke, it is important to take a wider perspective and include other costs, such as rehabilitation costs, informal caregiver costs and indirect costs, to be able to detect a possible cost shift. An intervention that is cost-effective for the hospital might put a larger financial burden on the nursing home, rehabilitation service or on the informal caregivers, the family and the society at large.

A comprehensive review collecting the available evidence on the cost-effectiveness of different integrated stroke care arrangements and their health outcomes can provide more insight into this issue. This review aims to provide an overview of the economic evidence of the currently existing types of post-emergency integrated care arrangements for stroke patients.

## Methods

### Study selection criteria

The current review is restricted to empirical studies that provide quantitative data, thus excluding qualitative studies, reviews and case reports. Only studies of stroke populations were included in which integrated care was studied, and where an economic evaluation was reported (either costs or resource use with mentioning of unit prices). The quality of the economic studies was evaluated by the researchers and studies of low quality were excluded from the final analysis (see Appendix).

**Integrated care arrangement:** To ensure we included all currently existing integrated care arrangements, we adopted the broad definition of integrated care of the European Pathway Association; ‘Integrated care is a multidisciplinary tool to improve the quality and efficiency of evidence based care which is used as a communication tool between professionals to manage and standardize the outcome orientated care.’ [3].

**Economic evaluation:** Primary studies that were either a full economic evaluation or a cost analysis were included, following the classification of economic studies of Drummond [8]. Studies that provided only a cost-outcome description, cost description or modelled costs were excluded. The review includes economic studies along the full spectrum of services––acute care, rehabilitation and long-term support.

### Literature search strategy

The databases of MEDLINE and EMBASE were searched in parallel for relevant English articles published up to July 11th, 2011. The search terms used for ‘economic evaluation’ were: economic analysis, economic evaluation, cost-benefit, cost-effectiveness, costs, health resources, resource utilization and utilization of health care. The search terms for ‘integrated stroke care’ were stroke service, stroke unit, integrated stroke care, organized stroke care, stroke rehabilitation, integrated care pathway, clinical pathway, critical pathway and disease management. Only articles that included both the economic information and the integrated stroke care element were examined further. Additional studies were identified through reference lists and overview articles.

The search strategy produced a total of 415 articles. After removing 150 duplicates, a further 179 articles were removed after screening titles. Screening abstracts and full-text led to the removal of 68 more articles (including eight articles from which we failed to obtain the complete article [9–16]). Four studies were considered to be of low quality, and were therefore excluded from the final analysis [17–20] (see Appendix). This left a total of 15 studies for inclusion in the final review.

### Data extraction and synthesis

Two researchers (JT and AS) independently selected studies; any disagreement was resolved through consensus. One reviewer (JT) extracted information from the selected studies.

Given the heterogeneity of the included studies, a qualitative approach summarizing the characteristics and results of the selected studies was used for data synthesis. The results of the studies are not pooled quantitatively, given the variation in study characteristics and methodology.

From the articles, information was extracted on study type, intervention details, control details, number of subjects, length of follow-up, type of facilities included in the cost analysis, type of economic study, costing perspective, mean cost per patient, type of cost included, how costs were determined and whether the authors had conducted sensitivity analysis. The direction and magnitude of total costs per patient in the intervention relative to the comparator were tabulated.

Several studies that reported a cost analysis had published health outcomes of the trial elsewhere. To provide a comprehensive overview, the health outcomes of these studies were also included in the final results table in this review.

The level of evidence concerning the cost-effectiveness for each service type was determined subjectively by weighing the number of studies, the consistency of cost trends, the ‘robustness’ of the results and the methodological quality of the study.

### Intervention categories

The interventions reported in the fifteen studies were grouped into four categories, which were deduced from the material; 1) early supported discharge (n=6), 2) home-based rehabilitation (n=4), 3) stroke unit care (n=2), and 4) stroke service from acute to chronic phase (n=3). The intervention categories are associated with different parts of the rehabilitation process, although there can be some overlap between interventions. For example, some interventions that focus on early-supported discharge also include home-based rehabilitation, or rehabilitation in a day-hospital. The economic evidence will be presented separately for each intervention category.

A short description of characteristics of each intervention category is provided here. In early-supported discharge, a multidisciplinary team facilitates discharge in order to reduce the acute hospital stay [21, 22]. In home-based rehabilitation, coordinated multidisciplinary rehabilitation takes place in the house of the patient [23]. In a stroke unit, care is organized by using clinical pathways for diagnosis, treatment, prevention of complications and rehabilitation specifically for stroke care. In addition, multidisciplinary teams coordinate care, rehabilitation therapy and patient education aimed at reaching predefined goals before the patient is discharged [24]. A stroke service was defined by one study as ‘a network of service providers, working together in an organized way to provide adequate services in all stages of the follow-up of stroke patients [25]. It includes a hospital stroke unit and at least one other service provider.

## Results

### Study characteristics

Ten out of fifteen studies were set in Europe (of which five were in the UK) [22, 25–33], two in Australia [34, 35] and Canada [36, 37], and one in Hong Kong [38] (Table 1). The time horizon of the studies was generally short; most of the studies followed the subjects for a year (n=9), and the rest for a shorter period (n=6). Most (n=12) of the included studies were randomized controlled trials, and three were non-randomized. The study size ranged from 83 to 598 subjects.

Even though 11 out of the 15 included studies were published after 2000, for all but one study [37] the data were collected from trials performed before 2000.

The intervention and the comparators are described in detail in Table 2 and the economic characteristics of the included studies in Table 3. Six studies were classified as ‘cost analysis’, seven studies as ‘cost-effectiveness study’ and two as ‘cost minimization analysis’ (although some were labelled differently by their authors). The scope of costs included varied, with only four studies [31, 32, 34, 37] using the preferred societal perspective, and the rest (n=11) adopting a health care perspective, including only costs related to the health care system.

### Economic results

In this section, an overview is presented of the economic results per intervention category (Table 4).

#### Early-supported discharge

In many places, early discharge from the hospital is widely practiced and encouraged. However, the degree to which discharge is facilitated or aftercare is arranged, differs greatly between settings [39]. The six studies reporting on the costs of early-supported discharge investigated slightly different versions of early supported discharge services. The interventions have in common that all included at least a multidisciplinary team, and they all aimed to reduce the length of acute-hospital stay.

For all six studies, the intervention did not result in worse health outcomes when compared to the comparator. Early-supported discharge combined with home rehabilitation even resulted in a significantly higher SF-36 score in the study of Teng et al [36].

Six out of six studies showed that early-supported discharge resulted in lower costs, in the range of a 4–30% cost reduction compared to usual or conventional care, but only in the study of Teng et al. [36], this difference was significant. Anderson et al. [34] report that although the cost reduction of 20% was not significant, sensitivity analysis found consistently lower costs for the intervention. Both Anderson [34] and McNamee [27] reported that the costs of the remaining care at home significantly correlated with a patient’s level of disability; those with mild disability scores had significantly lower costs compared to those with moderate disability scores, even after adjustment for age, co-morbidity, and presence or absence of a caregiver.

Beech et al. [26] considered it unlikely that early-supported discharge leads to financial savings, but due to a shorter length of stay it might release capacity for an expansion in caseload, which can also be a desirable outcome. In support of this hypothesis, both McNamee et al. [27] and Van Koch et al. [28] reported a significant reduction in the length-of-stay, which most likely explains the reported reduction in costs.

#### Home-based rehabilitation

The four studies that compared home-based rehabilitation were set in the UK [29, 30], Canada [37] and Sweden [33]. Gladman et al. [29] and Andersson et al. [33] both compare home-based rehabilitation to hospital-based rehabilitation, Roderick et al. [30] compared the home-based rehabilitation to care in a geriatric day-hospital, and Markle-Reid et al. [37] compared the delivery of home-care by a specialized team to usual home care. All the home-based care interventions have in common that the care at home was delivered by a multidisciplinary team.

Three out of four studies [29, 30, 37] reported non-significant higher costs for the intervention, accompanied by a significant improvement in the Barthel Index for Roderick et al. [30] and a clinically important improvement in the SF-36 score in the intervention group reported by Markle-Reid [37]. In the study of Gladman et al. [29] younger patients seemed to benefit more from home-care and older patients from hospital-based care.

In contrast to the three other studies, Andersson et al. [33] reported similar costs for home-based rehabilitation compared to hospital rehabilitation. The authors of this study suggested that home-based rehabilitation might in fact turn out to be costs-saving since most patients who should receive home-based rehabilitation had to wait longer in the expensive acute care beds of the hospital compared to the hospital-based group, because the necessary adjustments had not taken place in their houses. The authors suggest that a more smooth transition will render home-based rehabilitation cost-effective.

Home-based rehabilitation is often presented as a less expensive alternative for inpatient rehabilitation, as expensive traditional hospital care is substituted by less expensive care in the patient’s home [33, 37]. The results presented above, however, suggest the opposite. Possible explanations for the lower costs of inpatient care are a smaller number of staff involved, and the gaining of scale economies [29]. Such advantages were not available to the home-based rehabilitation investigated in these studies.

Roderick et al. [30] report that even though health care costs were lower for the home-based rehabilitation group, this was offset by higher social service costs. Andersson et al. [33] reported a similar cost-shift from health care providers to social welfare providers in the home-based rehabilitation group. A narrow costing perspective, looking only at costs for the health care providers may therefore paint a misleading positive financial picture.

The higher costs for the specialized team approach for home-based care in the study by Markle-Reid et al. [40] can be explained more straightforward; the team-approach had higher per-person costs of health service use compared to conventional home-care. The intervention did not pay for itself by reducing the use of expensive health care resources as the authors had expected.

From these four studies it seems likely that home-based rehabilitation after discharge will be cost-neutral from a societal perspective, and better quality of care could be achieved at similar costs.

#### Stroke unit care

The two studies evaluating stroke units set in Australia [35] and the UK [32]. In both of the studies, stroke units were compared to other types of interventions that could be used during the post-emergency phase of stroke care. Moodie et al. [35] compared three ways of providing in-hospital stroke care; 1) stroke unit care, 2) conventional in-hospital care, and 3) stroke care provided by an in-hospital stroke team. Patel et al. [32] also compared three interventions in the post-emergency phase; 1) stroke unit care, 2) general ward with a stroke team, and 3) stroke management at home. Both studies calculated incremental cost-effectiveness ratios (ICER) for all interventions.

In the study by Moodie et al. [35]; the costs of stroke unit care were 26% more than conventional in-hospital care, which was borderline significant (p=0.08). However, the health outcomes of stroke unit care were better than for conventional care. A more thorough adherence to process indicators and a decreased complication rate were found [35]. The incremental cost-effectiveness ratio (ICER) of stroke unit care over conventional care was AUS$9867 per patient achieving thorough adherence to clinical processes and AUS$16,372 per patient with severe complications avoided. The authors therefore concluded that dedicated stroke unit care was cost-effective. An in-hospital stroke team was the most costly intervention, and did not yield additional health gain. The study however only included patients with typically moderate stroke; and the results might not be applicable to the entire stroke population [35].

In line with the findings of Moodie et al. [35] stroke unit care was the most expensive of the three interventions in the study by Patel et al. [32], but also achieved better health outcomes. In-hospital stroke team care was dominated by stroke management at home, which was both more effective and less costly. Compared to stroke management at home, the ICER of stroke unit care was £682 of avoiding 1% of death and institutionalizations, or £89 132 per quality-adjusted life year (QALY) gained. These results should be interpreted with caution; Patel only included patients with a moderately severe stroke, who could be supported at home.

The two studies reported here yielded very similar results, and provided moderate evidence that at least for in-hospital care and for patients with a moderately severe stroke, care in stroke units yields better health outcomes. These improved outcomes come at a higher cost compared to stroke management at home, conventional in-hospital care or in-hospital stroke teams.

#### Stroke service

The stroke services in the three studies that we included differed in their degree of service integration. The study by Van Exel [25] for example studied three complete stroke services, while the stroke service of Claesson [31] mainly included a hospital and geriatric care component, meant for older patients. Fjaertoft [22] focused on improved transitions between acute care, early-supported discharge from a stroke unit and the primary care services with home care.

The stroke service described by Claesson et al. [31] only included patients older than 70 years, who had not been in a nursing home at the time of stroke. Their stroke service resulted in a non-significant cost reduction of 11% after the first year, with similar health outcome as the care as usual group. This study found a large variation in costs per patient, which was related to stroke severity at onset. Due to sample size restrictions it was not analyzed whether the stroke service was more cost-effective for certain patient groups based on stroke severity.

Fjaertoft et al. [22] observed a similar cost reduction of 13%. The patients who received care via the integrated stroke service also had a significantly higher Rankin scale (65 vs. 52% independence; p=0.02), Barthel index (60 vs. 50% independent in ADL; p=0.05) (41) and higher Quality-of-Life (mean score 78.9 vs. 75.2; p=0.05) [22].

Van Exel [25] evaluated three different stroke services in the Netherlands. The services all had different organizational characteristics and included different elements. The stroke service in Delft was consistently characterized as the most organized, and with the most clear-cut patient trajectory. Compared to usual care, the stroke service in Delft was also the only one of the three stroke services that showed a minimal reduction (5%) in costs together with a better health outcome. The stroke service in Nijmegen cost 50% more, at similar health outcomes. The stroke service in Haarlem was 22% more expensive, with worse health outcomes.

Two out of three studies reported a cost reduction, against similar or better health effects. The large differences in the organization of the stroke services in the three studies can explain the different results in both costs and outcomes.

## Discussion and conclusions

This review investigated economic evidence for four types of integrated care arrangements. In conclusion, six out of six studies on early supported discharge reported reduced costs with similar (n=5) or better (n=1) health outcomes. Apart from possible cost-savings, early supported discharge has the potential to free-up capacity for acute care. Home-based rehabilitation after stroke is most likely cost-neutral from a societal perspective, and can lead to improved quality of life. Two studies provided evidence that the use of stroke units yields better health outcomes, but at a higher cost compared to conventional in-hospital care or mobile stroke teams. The three studies that reported on integrated stroke services studied services that were substantially different from each other. Nonetheless, the trend in the results suggests that service integration can indeed be cost saving for stroke care.

Our findings differ at some points from an earlier review of economic evidence on stroke rehabilitation [42]. That review concluded that the costs of care in a stroke unit were comparable to care in another hospital ward, while we found higher costs [32, 35]. For home-based rehabilitation, the authors of the review concluded that studies showed conflicting results. For ESD however, their findings were in line with ours. We recognize that for integrated care arrangements, it might be true that ‘*the devil is in the details*’, meaning that two seemingly similar programs can have very different effects because of differences in context (e.g., size of the hospital), or implementation (e.g., culture differences). An evaluation of integrated care should therefore take those variables into account. We attempted to tackle this issue by providing detailed information about the precise intervention and the setting, for as much as it was described in the articles.

### Implications for future research

More evidence is needed to determine if and which integrated care arrangements are cost-effective for stroke patients. For future research, we recommend to take the following key points into account:

#### Include stroke severity as variable

From both a clinical and rehabilitation perspective, it makes sense to divide the stroke population into three groups; those who are mildly, moderately and severely affected. Division in these groups allows for the optimal use of resources [43]. For those who are mildly affected, early supported discharge and rehabilitation at home will probably be sufficient for recovery. For those severely affected, intensive rehabilitation programs in a rehabilitation centre might not have sufficient effect, and this group might be best off in a nursing home. The middle group, those who are moderately affected are the most interesting from a cost-effectiveness perspective. Specifically for this group of patients, it is important to find out which type of rehabilitation care (e.g., at home, in a rehabilitation centre or in a nursing home) yields the best health effects at the lowest costs. Since stroke severity is such an important variable in the outcome and effectiveness of stroke interventions, we therefore recommend that 1) future research should take stroke severity into account when researching cost-effectiveness and 2) cost-effectiveness studies for stroke interventions should focus on the patient groups with moderate and severe stroke.

#### Adopt a societal costing perspective

A variety of costing perspectives were used in the included studies. Besides creating a problem for comparison, this also poses a problem for the reliability of the findings; omission of certain costs can greatly influence the relative cost-effectiveness. When a health care perspective is used for example, the indirect costs and costs to informal caregivers are not included. A home-based intervention might then save money to the health care system, but put additional financial strain on the informal caregiver. We therefore strongly recommend that informal caregiver costs are included and a societal approach is adopted to assess the real cost-effectiveness of an intervention.

#### Include long-term costs and effects of treatments

The follow-up in the included studies was maximally twelve months after stroke onset. However, some late complications of stroke, such as depression only appear after one to two years. To ensure that all relevant costs and effects of interventions are included, we therefore recommend adopting a longer time horizon in cost-effectiveness studies for stroke interventions.

#### Adopt uniform measurement instruments

In order to compare studies, it is also advised to have more uniformity in the use of measurement instruments for health outcomes, e.g., Quality of Life and (social) participation scales.

Lastly, it was striking to see that even though some studies were published after 2005, all data analyzed were collected before 2000. It is questionable whether the results of these studies reflect current medical practice. We therefore recommend that new cost-effectiveness studies will be performed, investigating up-to-date ways of delivering integrated stroke care.

## Reviewers

**Thomas Jeerakathil,** Associate Professor of Neurology at the University of Alberta in Canada

**Martien Limburg,** neurologist, Professor, Flevoziekenhuis Almere, the Netherlands

**Silvina Santana**, PhD, Associate Professor with Agregação, Institute of Electronics Engineering and Telematics of Aveiro, Department of Economics, Management and Industrial Engineering, University of Aveiro, Portugal

## Figures and Tables

**Table 1.**
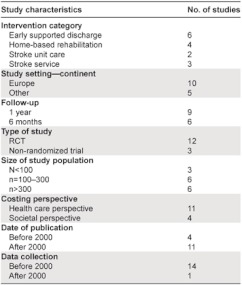
Summary of study characteristics.

**Table 2.**
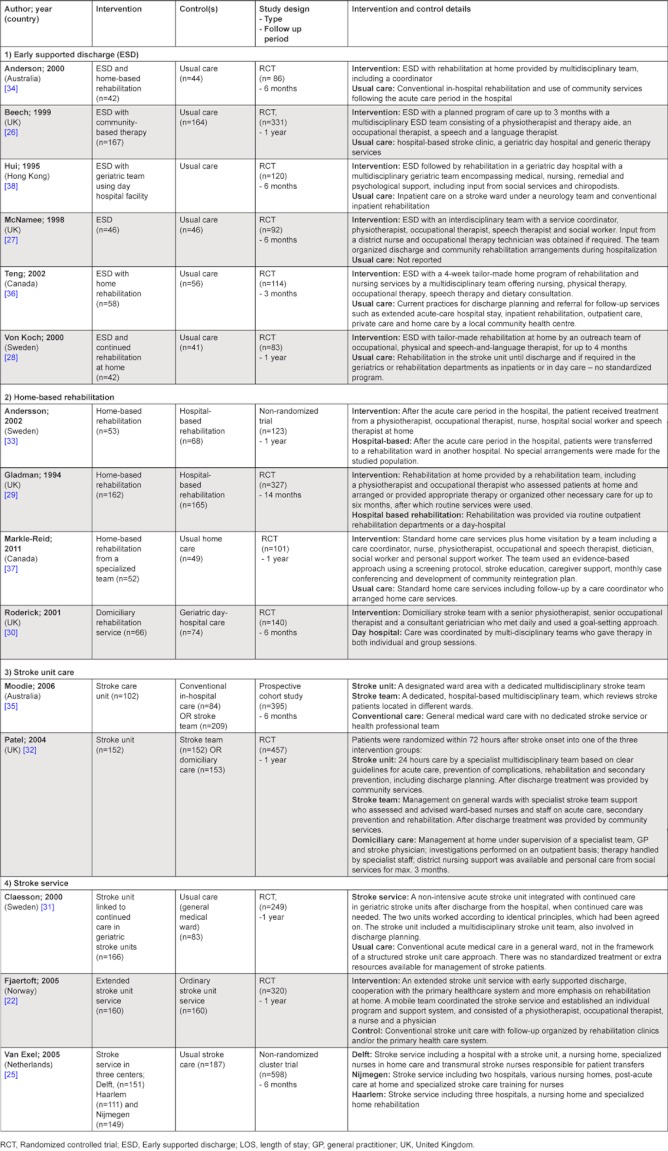
Study characteristics, intervention and control details.

**Table 3.**
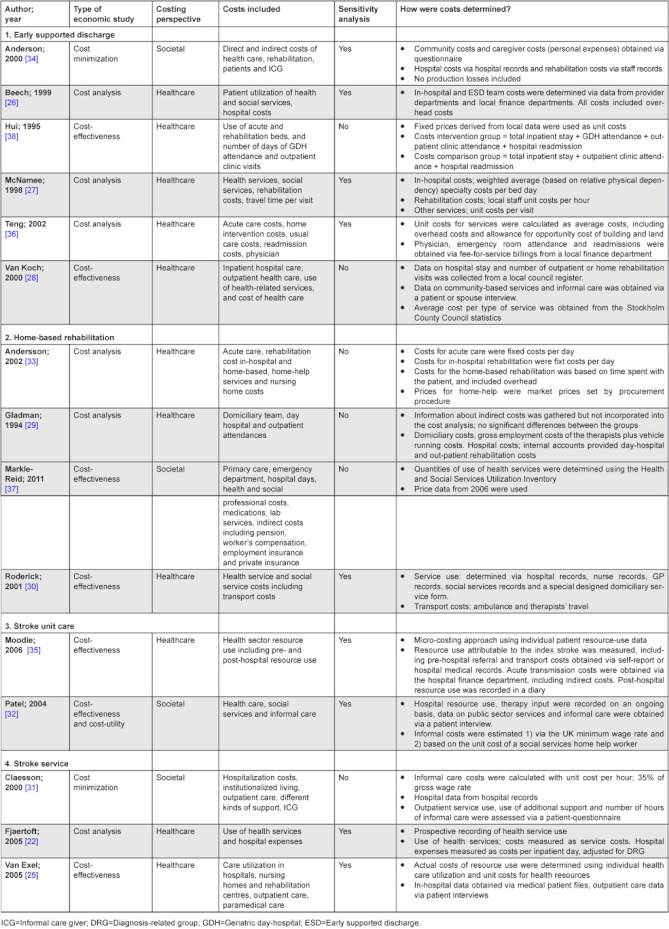
Characteristics of the economic analysis.

**Table 4.**
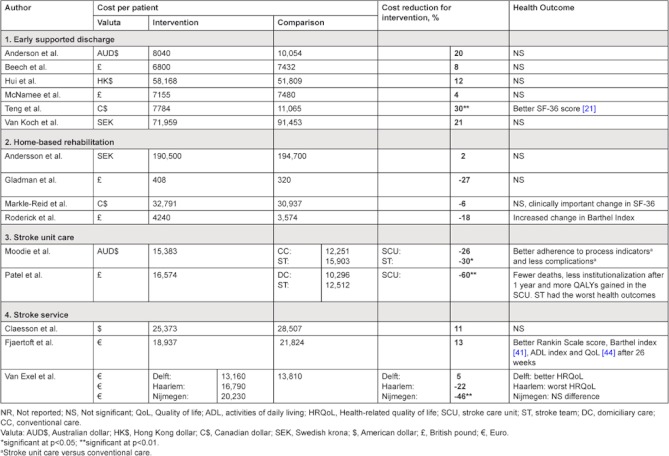
Comparison of costs and health outcomes reported by intervention category.

**Appendix:**
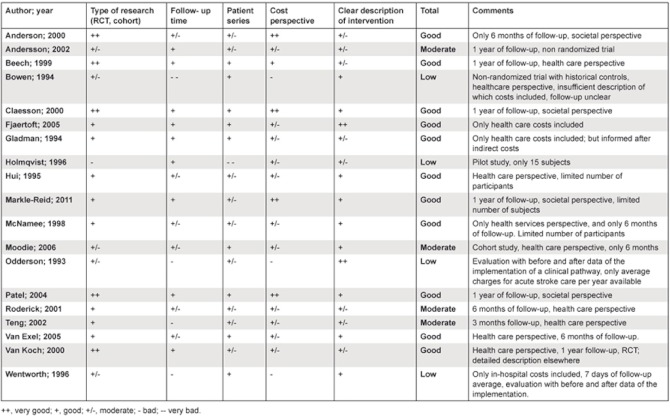
Methodological quality of the studies.
